# A qualitative exploration of menstruation-related restrictive practices in Fiji, Solomon Islands and Papua New Guinea

**DOI:** 10.1371/journal.pone.0208224

**Published:** 2018-12-03

**Authors:** Yasmin Mohamed, Kelly Durrant, Chelsea Huggett, Jessica Davis, Alison Macintyre, Seta Menu, Joyce Namba Wilson, Mary Ramosaea, Michael Sami, Dani J. Barrington, Donna McSkimming, Lisa Natoli

**Affiliations:** 1 Burnet Institute, Melbourne, Victoria, Australia; 2 Department of Epidemiology and Preventive Medicine, Monash University, Melbourne, Victoria, Australia; 3 WaterAid, Melbourne, Victoria, Australia; 4 Plan International, Buka, Autonomous Region of Bougainville, Papua New Guinea; 5 Susu Mamas, Goroka, Eastern Highlands Province, Papua New Guinea; 6 International Planned Parenthood Federation, Sub-Regional Office for the Pacific, Suva, Fiji; 7 School of Civil Engineering, University of Leeds, Leeds, United Kingdom; 8 International Women’s Development Agency, Melbourne, Victoria, Australia; London School of Hygiene and Tropical Medicine, UNITED KINGDOM

## Abstract

Attitudes and beliefs about menstruation can place restrictions on menstruating women and girls, limiting their ability to fully participate in community life, education and employment. This paper presents evidence on menstruation-related beliefs contributing to restrictive practices in Papua New Guinea (PNG), Solomon Islands (SI) and Fiji. Focus group discussions and interviews were undertaken with 307 adolescent girls, women and men in a rural and urban site in each country. Data were analysed using an inductive thematic approach. Participants described a range of attitudes and beliefs that restrict the behaviour of menstruating women and girls. Themes include the belief that menstrual blood is ‘dirty’; that when menstruating, girls and women can bring ‘bad luck’ to men; secrecy and shame associated with menstruation; and beliefs about the impact of certain behaviours on menstruation and health. Restrictive practices were more frequently reported in PNG and SI than Fiji, and more common in rural compared with urban sites. Some restrictions, such as avoidance of household chores, were perceived as desirable or driven by women themselves. However participants identified other restrictions, such as not being able to attend church or hygienically wash menstrual hygiene materials, as unwanted, in some cases impacting on participation in school, work and community life. Education initiatives guided by women and girls, implemented by local stakeholders and grounded in a sound understanding of specific contexts are needed to address discriminatory attitudes and beliefs that contribute to unwanted restrictions, and to support enabling attitudes and beliefs regarding menstruation.

## Introduction

In order to manage menstruation effectively and hygienically, girls and women need access to information about the menstrual cycle and how to manage it effectively; clean, affordable, absorbent materials that can be changed in privacy as often as necessary for the duration of a menstrual period; soap and water for washing the body as required; and facilities to dispose of used menstrual management materials [1]. This is collectively described as adequate menstrual hygiene management (MHM). However for many women and girls, especially those in low and middle-income countries, menstruation presents numerous challenges. These include: discriminatory attitudes, beliefs, social norms and taboos relating to menstruation; lack of access to affordable and effective menstrual hygiene materials; and lack of access to appropriate water, sanitation and hygiene (WASH) facilities [2–7].

Globally, attitudes, beliefs and social norms relating to menstruation vary widely, and these variations impact on practices during menstruation. For example, people in some settings believe that menstruation is dirty and that menstruating women are unclean [2, 8, 9]. Beliefs such as these can contribute to ‘restrictive practices’. For the purpose of this paper, we define a ‘restrictive practice’ as any kind of restriction that is placed on menstruating girls and women (either self-imposed or imposed by others), and is influenced by prevailing socio-cultural, religious or traditional beliefs and norms. Restrictions may include diminished mobility, seclusion, dietary restrictions, or being prevented from fully participating in community life [10]. Cultural perceptions and restrictive practices associated with menstruation can serve to isolate and stigmatise girls and women [11, 12]. Where this is the case, stigma and silence around menstruation can contribute to gender inequality that discriminates against women and girls throughout the lifecycle [13].Such restrictive practices can also influence MHM and the extent to which menstruation can be managed effectively and with dignity [4, 5]. Although robust evidence is lacking, unhygienic MHM materials may contribute to skin irritation and reproductive tract infections [14–16].

Restrictive practices during menstruation can have considerable psychosocial implications for girls and women. These are likely to be understood and experienced differently by individuals [5], and in some cases such restrictions are perceived as positive by women and girls, for example providing welcome opportunities to rest and spend time with other women [17]. More commonly however, the available evidence reports the limitations menstruation-related restrictions place on women and girls’ ability to fully participate in community life, education and employment [4]. In some cultures, menarche signifies readiness for sexual activity and marriage, with particular implications for sexual and reproductive health as well as educational attainment [18–20]. The onset of menstruation often brings with it new expectations for how girls should behave and interact with others, particularly boys and men. When menstruating, girls may be expected to stay away from their peer group, avoid male community members, and not visit certain locations, such as churches [12]. In some contexts, including in the Pacific, menstrual taboos and norms direct girls and women to avoid cooking or eating certain foods, and in some cases, to avoid bathing during menstruation [21].

Mobility restrictions, lack of facilities to manage menstrual bleeding at school, fear of leakage or staining clothes, and harassment by male students and teachers are demonstrated deterrents to girls attending school both globally [12, 22, 23] and in the Pacific [24]. Prolonged periods of reduced participation and absenteeism can contribute to school drop-out and reduced educational attainment [12], with long-term consequences for economic and health outcomes [25–27].

Women in workplaces also face a range of challenges managing menstruation at work, including lack of time or facilities to change MHM materials [28], humiliation and embarrassment [5], which may influence women’s participation in the workforce. Reduced workforce participation affects women’s individual and household income [28, 29], and has a demonstrated effect on country-level economic growth [30, 31]. At present however, evidence of the impact of poor MHM on women’s economic participation is lacking.

The majority of the available evidence on menstrual health comes from studies conducted in Asia and Africa, and there is limited published literature on the challenges and barriers to managing menstruation in the Pacific. Due to the distinct and diverse cultures and geographies found within the Pacific, it is likely that the challenges faced by women and girls in this region differ from those in Asia and Africa. To address this gap, we undertook a formative study to inform MHM programming in Fiji, Papua New Guinea (PNG) and Solomon Islands (SI). These three countries were chosen to provide a snapshot into the barriers to effective MHM in Melanesia. This is the first qualitative multi-country study on menstrual hygiene in the Pacific, and importantly includes the perspectives of predominantly underrepresented groups in the MHM literature including women in informal employment, girls not attending school and women living with a disability.

The purpose of this paper is to describe menstruation-related attitudes and beliefs that contribute to restrictive practices in PNG, SI and Fiji; the impact of these restrictions on the lives of women and girls; and associated implications for programming. The findings presented here are part of a broader study to explore the challenges experienced by women and girls in managing their menstruation, and whether these challenges make it hard for them to equally participate in school, work and community life.

## Methods

### Study setting

The study was conducted in one urban and one rural community in each of the study countries. SI, PNG and Fiji are culturally diverse Pacific Island nations with strong Melanesian ancestry. The majority of people in PNG and SI identify as Christian [32, 33]; over 60% of Fijians are Christian and the majority of Fijians of Indian descent (comprising 37% of the population) are Hindu [34]. SI and PNG face significant economic and human development challenges [35, 36]. Fiji by comparison has made greater advancement in economic and human development indices [37]. As a region, the Pacific has some of the lowest WASH coverage in the world. Access to basic sanitation services, such as improved toilets not shared between households, is particularly poor in PNG (55% urban and 13% rural) and SI (76% urban and 18% rural) [38]. Gender inequality is a critical development issue in each of the three countries, and women and girls in the Pacific experience some of the highest rates of gender-based violence in the world [39].

### Study design

For this formative qualitative research project, we undertook focus group discussions (FGDs), in-depth interviews (IDIs) and key informant interviews (KIIs) with a purposive sample of adolescent girls, women and men.

The content of FGD and interview question guides was informed by the Ecological Framework for MHM [40] and a review of relevant literature [41]. Topic areas explored socio-cultural norms, attitudes and beliefs related to menstruation, and how these influence the management of menstruation for girls and women. Question guides were developed by the study team in Melbourne and then reviewed and finalised in collaboration with the local research teams in each country. English question guides were translated into local language/s and back-translated into English to confirm accuracy.

FGDs included a number of participatory activities to stimulate discussion such as: body mapping, community mapping, the Ten Seed Technique [42] and drawing of an ‘ideal’ latrine [43]. All FGDs were sex-segregated with efforts made to ensure homogeneity of socio-economic status, community hierarchy and age (although this was often difficult in practice) to ensure that all FGD participants felt comfortable to openly share his or her thoughts and opinions.

### Participant recruitment

One urban and one rural research site was selected in each country based on pre-existing relationships between the local research partners and these communities. Local researchers identified communities with whom their previous engagement ensured a high level of trust and allowed them to easily identify participants from each of the predefined participant groups: adolescent girls / young women attending and not attending school; women working in formal and informal work settings; men; girls/women identified as being particularly vulnerable; vendors selling sanitary products; employers; teachers; health workers; and community leaders. These participant groups were selected in order to gauge a wide range of community perspectives on menstruation and related challenges and restrictive practices. A Community Engagement Officer from the local research team visited communities at each site to explain the objectives and proposed methodology of the research and gain permission from community leaders to undertake the project.

Individual participants were identified and invited to participate in interviews or FGDs once community permission was gained. Following community engagement processes (explained below) individuals were then selected on the basis of their availability and willingness to consent and take part in the study. Community Engagement Officers identified one school, one formal workplace and one informal workplace in each site; all School Principals and Organisation Heads (for formal workplaces) agreed to participate. Participant Information sheets were then distributed to female students from one class and female employees in each workplace. Efforts were made to ensure that one FGD with older schoolgirls (aged 17–19) and one with younger schoolgirls (aged 14–16) was undertaken in each country. Due to the requirement for parental consent for adolescent girls attending school, only girls whose parents had signed the consent form and who agreed to consent themselves were able to participate in the FGDs. At formal and informal workplaces, individual women self-selected to participate in the project. The Community Engagement Officers and community leaders also identified young women not attending school and men from the community who were provided with information about the purpose of the project and the requirements for data collection. All young women and men who volunteered to participate were included in the FGDs. Participants for KIIs were recruited from participating schools, workplaces and communities, as well as local health centres and shops. The Community Engagement Officer also contacted local disabled person’s organisations to identify women living with a disability in each country and invite them to participate.

### Data collection

The research team collected data between October 2016 and March 2017, with staged data collection in SI, Fiji and then PNG. Local staff employed by local research organisations and bilingual in English and local language/s collected the data. Three of the authors (LN, YM and CH) provided three days of training to all data collectors on the background and purpose of the research, the principles of qualitative data collection including the roles of facilitators and note takers, use of participatory tools and ethical considerations. Data collectors conducted interviews and FGDs in local languages (SI Pidgin; Fijian or Hindi in Fiji; Tok Pisin in PNG) or English, according to participant preference, and all discussions were digitally recorded. Facilitators were always the same sex as FGD or interview participants. In some cases, data collectors had pre-existing professional relationships with study participants (such as in the case of interviews conducted with health professionals). Data collectors transcribed and translated audio recordings verbatim into English.

The local research team undertook all FGDs and interviews in private locations to maximise confidentiality. Teachers and girls attending school participated in data collection activities in classrooms or teachers’ offices; FGDs with women in formal workplaces and KIIs with employers took place in offices, meeting rooms or specifically hired conference rooms; FGDs with men and young women not attending school were conducted in specifically hired venues within or nearby the community; IDIs with vulnerable/marginalised women were undertaken in private rooms or spaces, and the remaining KIIs took place in locations identified by participants themselves for convenience and privacy.

The research team conducted a total of 31 FGDs, 8 IDIs and 34 KIIs across the three countries. FGDs lasted between 60 and 90 minutes, IDIs up to 60 minutes and KIIs between 30 and 60 minutes. The number of FGDs and interviews was limited to two or at most three with each participant group in each country (one in each site) due to logistical and financial constraints. A summary of participant groups and data collection methods are presented in Table 1.

**Table 1:** Overview of participant groups by data collection method and country.

Participant group	Data collection method	Country	Number of sessions	Participant details		
				Age (years)	Sex (f/m)	Number of participants
Adolescent girls / young women in school	FGD	Fiji	2	16–19	f	13
		SI	2	18–21		20
		PNG	2	13–26		21
Adolescent girls/young women not in school	FGD	Fiji	2	18–20	f	15
		SI	2	13–20		15
		PNG	2	16–29		13
Women in formal employment (workplaces with fixed hours such as offices and factories)	FGD	Fiji	2	23–52	f	20
		SI	2	20–53		23
		PNG	2	24–43		17
Women in informal employment (workplaces with less structured hours such as market places)	FGD	Fiji	3	22–61	f	20
		SI	2	24–50		15
		PNG	2	19–60		23
Men	FGD	Fiji	2	25–68	m	15
		SI	2	23–47		14
		PNG	2	25–70		21
Vulnerable or marginalised women^a^	IDI	Fiji	2	31–35	f	2
		SI	3	-^b^		3
		PNG	3	-^b^		3
Vendors	KII	Fiji	2	-^b^	f	2
		SI	2	-^b^		2
		PNG	2	-^b^		2
Employers	KII	Fiji	2	-^b^	f	2
		SI	2	-^b^		2
		PNG	2	-^b^		2
Teachers	KII	Fiji	2	-^b^	f	2
		SI	2	-^b^	1f/1m	2
		PNG	4	-^b^	f	4
Health workers	KII	Fiji	2	-^b^	1f/1m	2
		SI	2	-^b^	1f/1m	2
		PNG	2	-^b^	1f/1m	2
Community/religious leaders	KII	Fiji	2	-^b^, 58	m	2
		SI	3	-^b^	m	3
		PNG	3	-^b^	1f/2m	3
**Total participants**						**307**

FGD, focus group discussion; KII, key informant interview; IDI, in-depth interview; PNG, Papua New Guinea; SI, Solomon Islands.

^a^ Such as women living with a disability, experiencing violence, socio-economically disadvantaged; young mothers and young married girls.

^b^ Data not collected.

Every effort was made to keep ages similar within FGDs with adolescent girls, in order to ensure younger girls felt comfortable sharing their opinions. However, in practice this was difficult to control for, particularly among adolescent girls not in school in PNG. Once participants for these FGDs came to the site of data collection, the research team decided that it would not be ethical or feasible to turn them away; therefore FGDs with adolescent girls not in school in PNG included young women up to the age of 29 years.

### Ethical considerations

Ethical approval for the study was received from the Solomon Islands Health Research and Ethics Review Board, Fiji National Health Research and Ethics Review Committee, Fiji Ministry of Education Heritage and Arts, PNG Department of Education Research and Evaluation Steering Committee, PNG National Department of Health Medical Research Advisory Committee and the Alfred Hospital Ethics Committee (Melbourne, Australia). The research team obtained written parental consent for all schoolgirls as well as assent from the girls themselves. Written informed consent was obtained from all other participants.

### Data analysis

The authors (LN, YM and CH) undertook preliminary data analysis during fieldwork. This involved daily debriefing with the local research team where detailed field notes from data collectors were discussed and emerging themes identified. Researchers amended question guides with the addition of specific probes to draw out themes requiring further exploration; the overall topics within the guides were however not altered. Once data collection was completed, all data were transcribed verbatim into local languages from recordings and translated into English. Using an inductive approach, the senior author (LN) familiarized herself with the data by reading each transcript before developing and refining one coding framework for all three countries. The coding framework was tested and revised in collaboration with two members of the research team (CH for SI and Fiji; and YM for PNG) before the three authors (LN and CH for SI and Fiji; LN and YM for PNG) systematically applied the coding framework line-by-line using qualitative data management software NVivo 10 (QSR International) for PNG and Fiji, and manual coding for SI. The authors then organised these codes according to overarching themes [44] and examined similarities and differences among participant groups and across study sites (urban and rural and across countries). Data were triangulated between relevant participant groups as well as across study sites and countries. For example, between FGDs with girls in school, KIIs with teachers, and FGDs with women in formal/informal employment, to allow for triangulation of data between students and teachers, but also between young women and older women.

The themes explored in this paper emerged from the data and were not preconceived prior to data collection. Themes were identified based on study findings and were chosen for inclusion because of their contribution to understanding the menstruation-related restrictions placed on women and girls.

The team leader (LN) produced a summary of findings that was reviewed for accuracy by the local researchers and shared with communities and stakeholders at the conclusion of the data collection in country. Once formal data analysis had been completed using the transcripts, LN shared a detailed report with findings and recommendations at an in-country workshop, affording an opportunity for key stakeholders and local researchers to provide feedback.

## Results

### Participant characteristics

Three-hundred and seven participants were recruited (246 female and 61 male) from the following stakeholder groups: adolescent girls / young women attending and not attending school; women working in formal and informal work settings; men; vulnerable or marginalised women; vendors selling sanitary products; employers; teachers; health workers; and community leaders. As outlined in Table 1, the majority of participants were female (80%), and ages ranged from 13 to 26 years for girls in school, 13 to 29 years for girls not attending school, 19 to 61 years for women and 23 to 70 for men.

Participants described a range of attitudes and beliefs that restrict the behaviour of menstruating women and girls across the three countries. These attitudes and beliefs can be categorised into four overarching, and often interacting, themes: the belief that menstrual blood is ‘dirty’; the belief that menstrual blood and menstruating girls/women can bring ‘bad luck’ to men and boys; the shame and secrecy that surrounds menstruation; and beliefs about the impact of certain behaviours on menstruation, health and well-being. Overall, these attitudes and beliefs, and the consequent behavioural restrictions, were more pronounced in PNG and SI when compared with Fiji. We consider each of these themes in turn, describe the restrictive practices associated with the beliefs and attitudes identified, and explore the reported impact that such restrictive practices have on the lives of girls and women. An overview of the main themes and associated restrictive practices is presented in Table 2 by country and site.

**Table 2:** Overview of main themes identified and the associated restrictive practices reported by country and site (urban/rural).

THEME	Associated restrictive practice	COUNTRY/SITE (urban/rural)					
Underlying menstruation-related attitudes and beliefs		Fiji		Solomon Islands		Papua New Guinea	
		Urban	Rural	Urban	Rural	Urban	Rural
Menstruation is ‘dirty’	Involvement in household tasks such as food preparation and cooking	(✓)	(✓)	✓	✓	✓	✓
	Avoiding sexual intercourse	✓	✓	✓	✓	✓	✓
	Participation in religious activities	(✓)	(✓)	-	✓	-	-
Menstruation and menstrual blood bring ‘bad luck”	Living separately from / avoiding contact with men and boys	-	-	✓	✓	✓	✓
	Working in the garden	(✓)	(✓)	-	-	-	✓
	Washing of commercial pads prior to disposal	-	-	✓	✓	-	✓
Menstruation-related secrecy and shame	Secretive washing and drying of reusable MHM materials or secretive personal hygiene practices	-	-	✓	✓	✓	✓
	Withdrawal from community life	-	-	-	✓	-	✓
	Reduced participation in / attendance at school	✓	✓	✓	✓	✓	✓
	Reduced participation in / attendance at work	-	-	✓	✓	-	✓
Menstruation and health-related beliefs	Avoidance of specific food and drinks	✓	✓	✓	✓	-	✓
	Avoidance of swimming	-	✓	-	-	-	-

✓, Restrictive practice reported

(✓), Restrictive practice reported in specific cultural groups only

-, Restrictive practice not reported.

#### Menstruation is ‘dirty’

All participant groups in SI and PNG perceived menstrual blood and menstruating women and girls as ‘dirty’ and ‘unclean’. As a consequence of this belief, some women and girls were prevented from, or chose to refrain from, household tasks such as food preparation, cooking and housework while menstruating. Indo-Fijian women of Hindu faith held similar beliefs, and reported that menstruating women should not cook food for their husband if he is a priest, or touch food being taken to the temple.

When women in PNG and SI were asked how they felt about restrictions on daily living activities such as cooking and cleaning, attitudes were mixed. Some accepted the restrictions as part of traditional culture or customs, some welcomed the time away from household responsibilities, while others stated that being prevented from undertaking certain tasks was a *‘waste of time’* and could be incredibly *‘boring’*. Several female participants in urban SI reported a preference for not cooking or handling food while menstruating, seeing it as a break from chores. Women and girls in PNG also stated that these traditional restrictions on cooking and housework were becoming less common and were often not adhered to, particularly in urban areas.

Beliefs about menstrual blood being dirty were often interconnected with beliefs and fears about menstrual blood bringing bad luck to men and boys. In rural and urban settings in PNG and SI, participants commonly believed that food prepared or cooked by menstruating women is harmful to men and boys, causing them to age faster or making them sick.

They are dirty and you know they have a … cultural belief. They think that you make the men … and the male sibling in the house … you know the food you touch makes them sick and they get older quicker and they don’t have the strength to work, you make them weak so … they won’t be … like physically active in doing men’s work … that’s the belief. (KII Female health worker; urban PNG).

Men and women from all three countries described a traditional restriction on sexual intercourse during menstruation. It was unclear whether this restriction was adhered to in SI and Fiji, although some men in PNG reported that sex was still practiced when their partners were menstruating. There was also a suggestion by men in urban PNG that a woman should notify her partner if she is menstruating to avoid misunderstanding, so that the man knows why she is declining sex and does not *‘start beating’* her.

In SI and PNG the traditional restriction on sexual intercourse was generally linked to beliefs about women being dirty or unclean during menstruation and causing harm to men, although in PNG some men believed a woman was at higher risk of becoming pregnant when menstruating. Some men in Fiji believed that sex during menstruation should be avoided because of an increased risk of acquiring sexually transmitted infections. In Fiji, many respondents, including community and religious leaders, cited the Bible’s Book of Leviticus as a rationale.

It is written in the Bible- do not approach a woman during her uncleanliness period. (FGD Men, rural Fiji).

Some women in rural SI described not being able to attend church when they were menstruating because they were ‘dirty’; others suggested that church attendance is acceptable, as long as women sit at the back on a bench allocated for menstruating women and do not deliver Bible readings or take Holy Communion. It was unclear whether women imposed these restrictions upon themselves or whether other churchgoers or church officials dictated them.

Men in rural SI believed that not attending church while menstruating was a preference among women, relating to the challenges in managing bleeding without access to good quality sanitary products. However, women in rural SI commonly viewed not being able to attend or fully participate in church as an unwanted restriction, and there was a feeling of embarrassment at having to sit at the back of the church because that would make it obvious to others that they were menstruating.

One challenge is that when menstruating we are not allowed to attend church. I don’t feel very good about it. Most [Christian] women would feel the same. (FGD Women in informal employment; rural SI).

Indo-Fijian women of Hindu faith also described not being able to go to temple or pray at home while menstruating. This was described as an unwanted restriction for some, while others accepted it as part of their religious beliefs. One healthcare worker reported that Hindu women would take the oral contraceptive pill to delay menstruation in order to attend important religious ceremonies.

#### Menstruation and menstrual blood brings ‘bad luck’

Among participants in PNG and SI, exposure to menstruating women or menstrual blood was traditionally believed to cause bad luck for men and boys, negatively impacting their health and physical strength and ability to hunt, fish and play sports.

[L]ike when they [men] go for hunting, they will not kill anything, so they must keep away from the ladies (FGD Women in formal employment; urban PNG).

This belief traditionally restricted the mobility of menstruating girls and women, and male and female participants in PNG and SI mentioned that menstruating women were not supposed to *‘sit close to men’* or *‘walk in front of men’*, and should avoid places where men or boys gather. These mobility restrictions are still observed by some, especially in rural settings.

She [a menstruating girl] is not allowed to meet her boyfriend (FGD Girls out of school; rural, SI).

Areas where boys usually gather, we [menstruating girls] don’t go past that place (FGD Girls in school; rural, SI).

In some very remote parts of SI and PNG, women and girls reported staying in a separate women’s house (‘haus meri’) every month for the duration of their menstrual period, although this practice is increasingly uncommon.

For us in [the] South too, if when you are having your period, you will leave the village and go live alone and also you will not eat from the same pot as those in your home. (FGD Women in informal employment, urban SI).

Beliefs about menstruation being associated with bad luck intrude on other parts of girls and women’s lives. One female participant in rural PNG reported a belief that women should stay home from work altogether when they were menstruating due to their unavoidable proximity to men. Other study participants did not voice this perspective.

[W]e have a strong taboo here about menstruation where men are not allowed to stay close to women who are menstruating or even take food. When women are menstruating, the blood that comes out of their body gives out a really bad smell and especially women who are working amongst the men in the same office, it is not right due to the … taboo towards menstruation. Therefore women who menstruate should be given time off so they can stay home and rest. (IDI Female employer; rural PNG).

In PNG, some female participants said they must avoid work in the garden during menstruation.

She is not allowed to go to the garden because when women who are menstruating go to the garden, pigs destroy the garden. (FGD Girls in school; rural PNG).

A similar belief exists among women of Rabian culture (a small Fijian ethnic group with descendants from Kiribati; Rabian women in formal employment participated in FGDs at both the rural and urban sites in Fiji) who described not being allowed to pick fruit from plants because the plants would die. It was not clear from participants’ responses whether this was an unwanted restriction or how much impact it had on their lives.

Some women and girls in rural PNG reported needing to wash commercial pads before burning them because the fumes (contaminated by menstrual blood) would otherwise be harmful to men and the community. This practice was also described in SI, where some explained it as a measure to reduce smell, while others related it to ‘black magic’.

I wash it to remove the blood …in case when I throw it away … somebody … opens it and sees it …others will put a spell on it if anyone sees it …it’s called ‘arua’ …something like that. (FGD Girls in school; urban SI).

#### Menstruation-related secrecy and shame

Study participants reported barriers to their ability to hygienically manage their menstruation. In rural and urban settings in SI and PNG, women and girls spoke of drying cloths used for managing menstrual bleeding outside on a line underneath a larger piece of cloth (out of direct sunlight), while others are more secretive and dry cloths inside the house.

In SI the stigma around menstruation holds particular significance for male and female siblings, compelling girls to be very cautious and secretive when managing their menstruation in order to ensure their brothers do not see menstrual blood or cloths. This limits when and where girls can wash, change and dispose their MHM materials.

It’s not really a taboo but concerning custom–when we have our period our brothers must not see it. If they see you have your period and a stain on the clothes, you will give them money to show respect for disrespect because they see this. (KII Health worker; urban SI).

Women and girls in SI and PNG spoke of needing to be more secretive and discrete with their personal hygiene during menstruation, and many spoke of washing in the sea or taking a bucket of water to a private location to wash privately to avoid others seeing menstrual blood.

Sometimes we go to the sea to wash and rinse and then come to the tap stand to bathe ourselves. First the blood is cleaned in the sea so it is not seen at the tap stand. (FGD Women in informal employment; rural SI).

In PNG and SI, some female participants described staying home during their menstrual periods and avoiding interactions. This is often self-imposed and frequently relates to a fear of clothes being stained by menstrual blood or fear that others can smell menstrual blood.

I don’t like … sitting among people, even female too, I don’t like sitting with them, because they can really tell … really smell … and say [all] sorts of things. So to avoid that from happening … I don’t like sitting down and hanging out with them. (FGD Women in formal employment; rural PNG).

While some women and girls in rural parts of PNG are not allowed to work in the garden, one woman from rural SI described using the garden as a place to isolate herself from others while menstruating.

I feel frightened to go around and meet people because of the smell, so I usually go to the garden to stay away from people in the village. (FGD Women in formal employment; rural SI).

Particularly in SI and PNG, teasing and harassment of menstruating women and girls further exacerbated the shame and secrecy surrounding menstruation. Teasing and harassment by boys was reported by girls in all three countries but was much more common in PNG and SI. In some cases this had a detrimental impact on girls’ ability to participate fully in school. In PNG and SI, girls reported being distracted from their school work due to a fear of staining their clothes with menstrual blood, and some participants in PNG reported that harassment by boys had in extreme cases resulted in girls leaving school altogether.

This is a challenge where girls are teased and made fun of at school and sometimes girls leave school because of the way she is being treated because of her menstruation. (FGD Women in informal employment; rural PNG).

While teasing and harassment was less commonly reported in Fiji, some girls did describe harassment at school, linking it to bribes and absenteeism.

Oh they will tease us. And sometimes they will just come to you and will be like, ‘I know what you have in your bag’. And through that they take money. You give me this and I will keep it as a secret. For some girls they find it fun, but some they find it embarrassing and some of them don’t even come back to school for the next day. (FGD Girls not in school, urban Fiji).

Girls in Fiji also explained that the practice of ‘*spot checks*’ (random checks of school bags for items that are not allowed such as mobile phones) adds to this problem.

According to some participants, fear of teasing impacted on women’s equal participation in work, primarily through self-imposed avoidance of male colleagues due to a fear that colleagues will smell menstrual blood.

I think in the office as well like trying to work with your male colleagues. Sometimes you will feel like they will say you stink. You would not want to go closer to them and talk to them. You want to be kept on your own. (FGD Women in formal employment; rural PNG).

Teasing and harassment also limits social participation for some women and girls in PNG. Girls reported choosing not to play with their friends due to a fear of being made fun of if their clothes became stained with blood. Others indicated that one reason they chose to stay home for the duration of their period was a fear of leakage and the humiliation and embarrassment associated with this.

The men interviewed suggested that teasing was related to a lack of knowledge about menstruation among men and boys. Adolescent boys were not interviewed as part of this project, therefore it was not possible to further explore their perceptions regarding menstruation and menstruation-related teasing.

#### Menstruation and health-related beliefs

Attitudes and beliefs around the impact of specific behaviours on menstruation, health and wellbeing more broadly were identified in all settings. Some female participants stated that fish, meat and other proteins should be avoided when menstruating–believing that these foods would make the menstrual flow heavier, or cause it to be more ‘*smelly*’.

While beliefs leading to dietary restrictions were less common in Fiji, adolescent girls, women and healthcare workers commonly believe that drinking cold water would cause heavier blood flow. It was also perceived that bathing in cold water causes stomach cramps/pain.

I am not allowed to drink iced water. And sour things. I just heard it from my mother. She told me not to drink iced water because it will make me bleed more. And eating sour things causes heavy bleeding as well. (FGD Girls not in school, urban Fiji).

I always advise them not to bath in cold water when they are menstruating, but to always bath in hot water. (KII Health worker, rural Fiji).

Menstruation-related beliefs also impact on personal hygiene practices. In the rural site in Fiji, girls not attending school reported that they do not wet their hair or swim while menstruating, because then *‘they would not menstruate properly’*. While many viewed these restrictions as positive, believing it would keep them in good health, some indicated that not swimming in the sea was a disadvantage, as they were unable to go fishing when menstruating. Women of Rabian culture mentioned not combing their hair during menstruation due to a belief that it would fall out.

Some Hindu Indo-Fijians also reported that menstruating women are not allowed to bathe or leave the home after 6pm, and reusable cloths hanging outside must be brought inside before this time, otherwise evil spirits will make them sick. This time point has general cultural significance for Indo-Fijians, for example children are not allowed to play outside after this time, but for women the restriction is followed more strictly during menstruation.

## Discussion

Our study found that underlying attitudes and beliefs around menstruation contribute to behavioural restrictions among women and girls in PNG and SI, and to a lesser extent in Fiji. Fijian women and girls may have been less likely to experience and report menstruation-related behavioural restrictions due to social and cultural changes and a move away from traditional lifestyles or simply cultural differences in the way menstruation is perceived between countries. While restrictions in all three countries seem to be followed less rigidly in urban communities, some restrictions continue to impact on hygienic and effective management of menstrual hygiene materials, consumption of specific foods and in some cases participation in school, work and community life for women and girls.

The stigma and secrecy that surrounds menstruation, and the consequent shame and embarrassment, can impact on women and girls’ ability to hygienically wash and dry reusable absorbent materials [24, 45–48]. Some of the girls and women in this study who were reliant on reusable materials reported drying materials out of direct sunlight to avoid other people seeing them. These findings are consistent with the existing literature; a survey in Afghanistan found that almost 70% of adolescent female research participants dried their menstrual cloths in the shade [5]. Not being able to hygienically dry reusable menstrual hygiene products in direct sunlight may increase the risk of reproductive tract infections, although definitive evidence for this link is lacking [16]. Where high levels of stigma and secrecy impede women and girls’ ability to manage their menstruation with dignity, their basic human rights are compromised [13].

Other restrictions had less of a clear-cut impact on household-level life. As seen in previous studies in PNG, restrictions on cooking, preparing food and performing household chores were common [49]. These restrictions may be positive and welcome, or negative and unwanted, depending on individual circumstances. Some women and girls perceived the avoidance of cooking and housework as beneficial, offering a welcome break from these responsibilities.

Some restrictions placed on menstruating women were not routinely adhered to, particularly as traditional lifestyles become less common. In PNG for example, men reported still having sex with their menstruating partners. This finding is consistent with another study in PNG, where women reported still having sexual intercourse while menstruating despite the cultural taboo, often under pressure from their male partners [49]. This finding by Vallely et al. and the insinuation from men in urban PNG in our study that women can only refuse sex when menstruating is suggestive of more systemic gender inequalities in this setting, including high levels of violence against women.

Beliefs that menstruation is ‘dirty’ and that menstruating women and girls bring bad luck to men and boys has impacts on the social lives of female participants in PNG and SI, particularly those in rural areas. In SI, women described feeling unhappy at not being able to attend church when they were menstruating, and some Indo-Fijian women expressed similar views about not being allowed to visit the temple. Bodily excretions such as menstrual blood are often considered unclean in Hindu faith, and comparable notions of menstrual ‘impurity’ have also been reported in some Christian communities [5]. Not being able to fully participate in church activities in a context where religion is an important part of everyday life impedes women’s ability to participate fully in cultural and community life and reinforces adverse gender norms in which women are perceived as lesser.

The shame and stigma associated with menstruation, particularly in PNG and SI, occasionally led to women and girls choosing to withdraw from community life while menstruating. Avoiding men and boys was traditionally associated with beliefs that menstrual blood is ‘dirty’ and brings bad luck, however participants in this study reported that more recently such avoidance relates to poor access to good quality absorbent products and a consequent fear of smelling bad, leakage, staining clothes, and harassment. While women and girls may themselves decide to withdraw from community life and not participate in activities such as playing with friends, it is the underlying shame and stigma surrounding menstruation that prevents women and girls’ full participation, entrenching isolation and stigmatisation during menstruation. Evidence of the impact of menstruation-related shame and stigma is lacking, however literature from other disciplines suggests that stigma can have a significant detrimental impact on the lives of individuals and if not adequately addressed can impede the effectiveness of programmatic solutions [50].

In rural parts of PNG and SI, some women and girls stay in separate houses for the duration of their menstruation. While this may impede women and girl’s ability to participate in community life, findings from a study in Eastern Indonesia suggest that living separately from men while menstruating can also be considered an important opportunity to spend time with other women and to rest [17]. The findings from our study did not conclusively indicate the impact of this traditional separation of menstruating women.

Our study finds that negative attitudes and beliefs around menstruation contribute to feelings of shame and embarrassment that can impact on participation in school. For example, teasing and harassment of menstruating girls was described in all three countries. Participants suggested that this contributed to absenteeism from school and some described reduced active participation in class activities for fear of being harassed. Adolescent girls appeared to be particularly vulnerable to teasing by boys at school. A study among schoolgirls in rural Kenya also found that harassment by boys–and in some cases male teachers–was common, adding to feelings of fear and shame [47]. Schoolgirls in Ethiopia, Tanzania, South Sudan and Zimbabwe also report being teased, humiliated and harassed by boys at school during menstruation [12]. The available evidence suggests a correlation between poor MHM and school absenteeism, however robust data on the effectiveness of MHM interventions on attendance at school are lacking [51–53]. Regardless of the impact on school attendance, teasing and harassment can have serious implications for mental health and gender equality [4, 13, 54].

Fear of staining and leakage was also reported to impact on girls’ attendance at school on days of heavy bleeding and potentially affect their ability to concentrate and participate in class. It is important to note that reduced participation and absenteeism from school is likely due to a range of factors including poor access to good quality absorbent materials, menstrual pain and inadequate WASH facilities, with stigma and shame being only one of the contributing factors [12, 22, 23].

The shame and secrecy associated with menstruation also had an impact on women’s participation in the workplace. Women in rural PNG described avoiding male work colleagues due to a fear of staining, leakage and the ‘odour’ associated with menstruation. Importantly, discriminatory attitudes towards menstruating women and girls were not the only factor affecting participation in employment. Particularly in rural PNG and SI, a lack of water and sanitation facilities that meet the needs of menstruating women and poor access to good quality sanitary products also reduced work attendance and meaningful participation. There is limited evidence on the quantitative and broader implications of inadequate MHM on participation in the workforce and the consequent economic impact of reduced attendance at work; further research into this area is warranted.

### Implications for programming

Behaviour change communication and community-level education integrated across a range of sectors have the potential to shift some of the discriminatory attitudes and beliefs that lead to unwanted behavioural restrictions [53]. Utilising key influencers such as community and religious leaders is critical for ensuring maximum reach and uptake [4]. The provision of accurate information about menstruation both in the community and in schools, targeting both students and parents, may also help to address underlying social norms around menstruation that impact on women and girls. Men and boys must be included in education efforts, as key change agents to tackling harmful gender norms and stigma linked to MHM. A particular focus on reducing teasing and harassment is important to include in school-based education for girl and boy students.

All programs aimed at improving women and girls’ health, education outcomes and economic opportunities could also incorporate an MHM component, taking into account pervasive cultural and social norms. Quality comprehensive MHM solutions call for effective community engagement approaches and multi-sectoral collaboration including gender, WASH, sexual and reproductive health, education and disaster response. In particular, it is imperative that local women and girls are meaningfully involved in any project impacting on MHM. As well as raising awareness and providing information about this important topic, programmatic approaches should also allow for women and girls to maintain their desired level of privacy when managing menstruation. This could be achieved through improvements in WASH facilities and increasing the accessibility of appropriate MHM materials, thereby reducing fears of leakage and smell. Reusable, internal products such as menstrual cups have been shown to be acceptable in Nepal and could be considered for the Pacific [55].

The restrictive practices highlighted by this study vary in public health and individual importance; this needs to be accounted for when considering the implications of the research for programming. For example, some restrictions (such as avoidance of iron or protein rich foods) may have a negative impact on physical health; some (such as restrictions on participation in religious events) may impact mental health or social inclusion, while other restrictions may be more benign in their impact (such as restrictions on combing hair); however all restrictions contribute to perceptions of women and girls as ‘other’ and lesser. When considering the implications of this research for programming interventions, it is sensible to prioritise addressing those restrictions having the most negative health or social impacts as well as those that are most important to women and girls. Such efforts need to be grounded in a solid understanding of contemporary local culture and context. What works in one setting will be considerably different to what works in another, and contexts are dynamic. Considering that not all behavioural restrictions are unwanted, and many are becoming less commonly practiced, a nuanced approach that engages local women and girls in program design, implementation and evaluations are needed.

Our study has several limitations. This research only took place in two sites in each country, and the cultural and ethnic diversity, differing expressions of gender inequality and the vast geographic spread of communities should be acknowledged when interpreting the findings. As a qualitative study, the results should be considered in context and not interpreted as being representative of the population as a whole. There is also a degree of sampling bias associated with convenience sampling which could have impacted on participants’ responses to our questions, potentially by exacerbating social desirability bias or alternatively by increasing the honesty and openness with which participants responded, due to the existing relationships with local research organisations. Identifying communities through these existing relationships with local researchers was the most practical approach to research in these contexts. Data collection did not occur to the point of ‘saturation’. Rather, the sample size (both the number of study sites and individual participants) reflects a pragmatic approach and the need to balance strong research with the financial realities of undertaking fieldwork of this nature. While every effort was made to include participants from similar age groups in each FGD, this proved challenging in practice and the broad age range in some FGDs may have impacted on participants’ desire to openly respond to questions, particularly for the younger girls or women. A further limitation is that we were not able to include all key stakeholder groups in this research project; in particular adolescent boys’ voices are missing from the narrative. This overall project aimed to understand how women and girls in PNG, SI and Fiji manage their menstruation, including the barriers and challenges, the impact on school and work, and potential opportunities for improvements. Due to the specific nature of the research aims, we were limited in our ability to comprehensively explore all menstruation-related cultural and social norms and taboos.

## Conclusion

Participants in PNG and SI, and to a lesser extent Fiji, reported social, cultural and religious beliefs and attitudes that contribute to behavioral restrictions for menstruating women and girls. These restrictions impact on their ability to manage menstruation effectively and with dignity, and fully participate in school, work and broader community life. While some restrictions may be desirable or self-imposed, others are unwanted and detrimental to the health and wellbeing of individuals and communities. The negative impacts of restrictive practices include exacerbation of shame and stigmatization, prevention from participating in religious activities, and potentially absenteeism from work and school. Further research is needed on menstruation-related restrictive practices in the Pacific, with a particular focus on the physical and psychological health and gender equality impacts. Deeper exploration of the quantitative impact of inadequate MHM on women’s participation in the formal and informal workforce would also be beneficial; for example lost productivity from menstruation-related absenteeism. Discriminatory attitudes and beliefs towards menstruation could be addressed through strengthening education programs in schools and communities more broadly. Women and girls must be central to the design and implementation of all menstruation-related interventions, to ensure that solutions are appropriate, acceptable and supportive of human rights.

## References

[1] Satterthwaite M. JMP Working Group on Equity and Non-discrimination Final Report. 2012.

[2] Burnet Institute, SurveyMETER, WaterAid Australia, Aliansi Rem aja Independen. Menstrual Hygiene Management in Indonesia: Understanding practices, determinants and impacts among adolescent school girls (Final report). Melbourne: Burnet Institute, 2015.

[3] Caruso B, Fehr A, Inden K, Sahin M, Ellis A, Andes K, et al. WASH in schools in Freetown, Sierra Leone: An assessment of Menstrual Hygiene Management in schools. 2013.

[4] Geertz A, Iyer L, Kasen P, Mazzola F, Peterson K. An Opportunity to Address Menstrual Health and Gender Equity. FSG, 2016.

[5] House S, Mahon Trs, Cavill S. Menstrual Hygiene Matters: a resource for improving menstrual hygiene around the world. UK: WaterAid, 2012 0968–8080.

[6] Tamiru S, Mamo K, Acidria P, Mushi R, Ali CS, Ndebele L. Towards a sustainable solution for school menstrual hygiene management: cases of Ethiopia, Uganda, South-Sudan, Tanzania, and Zimbabwe. Waterlines. 2015;34(1):92–102. 10.3362/1756-3488.2015.009

[7] Tjon A Ten V. Menstrual hygiene: a neglected condition for the achievement of several millennium development goals. Europe External Policy Advisors. 2007:1–22.

[8] Ahmed R, Yesmin K. Menstrual hygiene: Breaking the silence. 2008.

[9] Burrows A, Johnson S. Girls' experiences of menarche and menstruation. Journal of Reproductive and Infant Psychology. 2005;23(3):235–49. 10.1080/02646830500165846

[10] Trs Mahon, Cavill S. Menstrual hygiene matters: Training guide for practitioners. UK: WaterAid, 2015.

[11] Adhikari P, Kadel B, Dhungel S, Mandal A. Knowledge and practice regarding menstrual hygiene in rural adolescent girls of Nepal. Kathmandu University medical journal (KUMJ). 2006;5(3):382–6. 18604059

[12] Tamiru S. Girls in Control: Compiled Findings from Studies on Menstrual Hygiene Management of Schoolgirls. Addis Abbaba, Ethiopia: SNV Netherlands Development Organisation, 2014.

[13] Winkler. Taking the Bloody Linen out of the Closet–Menstrual Hygiene as a Priority for Achieving Gender Equality.

[14] Anand E, Singh J, Unisa S. Menstrual hygiene practices and its association with reproductive tract infections and abnormal vaginal discharge among women in India. Sex Reprod Healthc. 2015;6(4):249–54. Epub 2015/11/29. 10.1016/j.srhc.2015.06.001 . 26614609 10.1016/j.srhc.2015.06.001

[15] Das P, Baker KK, Dutta A, Swain T, Sahoo S, Das BS, et al Menstrual Hygiene Practices, WASH Access and the Risk of Urogenital Infection in Women from Odisha, India. PLoS One. 2015;10(6):e0130777 Epub 2015/07/01. 10.1371/journal.pone.0130777 ; PubMed Central PMCID: PMCPMC4488331. 26125184 10.1371/journal.pone.0130777 PMC4488331

[16] Sumpter C, Torondel B. A systematic review of the health and social effects of menstrual hygiene management. PLoS One. 2013;8(4):e62004 Epub 2013/05/03. 10.1371/journal.pone.0062004 ; PubMed Central PMCID: PMCPMC3637379. 23637945 10.1371/journal.pone.0062004 PMC3637379

[17] Hoskins J. The menstrual hut and the witch's lair in two eastern Indonesian societies. Ethnology. 2002:317–33.

[18] Glynn JR, Kayuni N, Floyd S, Banda E, Francis-Chizororo M, Tanton C, et al Age at menarche, schooling, and sexual debut in northern Malawi. PLoS One. 2010;5(12):e15334 10.1371/journal.pone.0015334 ; PubMed Central PMCID: PMCPMC3000342. 21151570 10.1371/journal.pone.0015334 PMC3000342

[19] Ibitoye M, Choi C, Tai H, Lee G, Sommer M. Early menarche: A systematic review of its effect on sexual and reproductive health in low- and middle-income countries. PLoS One. 2017;12(6):e0178884 10.1371/journal.pone.0178884 ; PubMed Central PMCID: PMCPMC5462398. 28591132 10.1371/journal.pone.0178884 PMC5462398

[20] Sommer M. Menarche: a missing indicator in population health from low-income countries. Public Health Rep. 2013;128(5):399–401. 10.1177/003335491312800511 ; PubMed Central PMCID: PMCPMC3743290. 23997288 10.1177/003335491312800511 PMC3743290

[21] CS WASH Fund. Menstrual Hygiene Management: Learning Brief from the Pacific Regional Learning Event. Civil Society Water, Sanitation and Hygiene Fund, 2016.

[22] Sommer M. Where the education system and women's bodies collide: The social and health impact of girls' experiences of menstruation and schooling in Tanzania. J Adolesc. 2010;33(4):521–9. Epub 2009/04/28. 10.1016/j.adolescence.2009.03.008 . 19395018 10.1016/j.adolescence.2009.03.008

[23] Unicef. Menstrual Hygiene in Schools in 2 countries of Francophone West Africa: Burkina Faso and Niger Case Studies in 2013 2013.

[24] Unicef, editor Solomon Islands: Incorporating MHM into national WASH in schools policies and guidelines. 4th MHM Virtual Conference, 22–23 October 2015; 2015.

[25] Gakidou E, Cowling K, Lozano R, Murray CJ. Increased educational attainment and its effect on child mortality in 175 countries between 1970 and 2009: a systematic analysis. The Lancet. 2010;376(9745):959–74. 10.1016/S0140-6736(10)61257-3 20851260

[26] Patton GC, Sawyer SM, Santelli JS, Ross DA, Afifi R, Allen NB, et al Our future: a Lancet commission on adolescent health and wellbeing. The Lancet. 2016. 10.1016/S0140-6736(16)00579-1 PMC5832967 27174304

[27] Women UN. Progress of the World’s Women 2015–2016. Chapter. 2015;2:71.

[28] Her+Project. Female Factory Workers’ Health Needs Assessment: Bangladesh. 2010.

[29] Water Supply & Sanitation Collaborative Council. Celebrating Womanhood: How better menstrual hygiene management is the path to better health, dignity and business. Malaysia: WSSCC, 2013.

[30] Klasen S, Lamanna F. The impact of gender inequality in education and employment on economic growth: new evidence for a panel of countries. Feminist economics. 2009;15(3):91–132.

[31] Organization for Economic Cooperation and Development (OECD), editor Gender Equality in Education, Employment and Entrepreneurship: Final Report to the MCM Meeting of the OECD Council at Ministerial Level; 2012; Paris: OECD.

[32] PNG National Statistics Office. Papua New Guinea 2011 National Report. Port Moresby, PNG: PNG National Statistics Office, 2011.

[33] Solomon Islands Government. 2009 Population & Housing Census: National Report. Honiara: Solomon Islands National Statistical Office, 2013.

[34] Fiji Bureau of Statistics. Population by Religion—2007 Census of Population Suva, Fiji: Fiji Bureau of Statistics,; 2016 [24 June 2016]. Available from: http://www.statsfiji.gov.fj/statistics/social-statistics/religion.

[35] UNDP. Human Developmnt Reports: Papua New Guinea 2016 [cited 2017]. Available from: http://hdr.undp.org/en/countries/profiles/PNG.

[36] UNDP. Human Development Reports: Solomon Islands 2016 [cited 2017]. Available from: http://hdr.undp.org/en/countries/profiles/SLB.

[37] UNDP. Human Development Reports: Fiji 2016 [cited 2017]. Available from: http://hdr.undp.org/en/countries/profiles/FJI.

[38] WHO, Unicef. Progress on drinking water, sanitation and hygiene: 2017 update and SDG baselines. Geneva: 2017 Contract No.: February 27.

[39] UN Women. Eliminating Violence Against Women in the Asia Pacific: it’s all of our responsibility. 2015.

[40] Emory University, Unicef. WASH in Schools Empowers Girls' Education: Tools for Assessing Menstrual Hygiene Management in Schools. 2013.

[41] Burnet Institute, WaterAid, International Women's Development Agency. The Last Taboo: Research on managing menstruation in the Pacific. 2016.

[42] Jayakaran R. The Ten Seed Technique People's Republic of China: World Vision, 2002.

[43] Connolly S, Sommer M. Cambodian girls' recommendations for facilitating menstrual hygiene management in school. Journal of Water Sanitation and Hygiene for Development. 2013;3(4).

[44] Pope C, Ziebland S, Mays N. Qualitative research in health care. Analysing qualitative data. British Medical Journal 2000;320:114–6. 10625273 10.1136/bmj.320.7227.114 PMC1117368

[45] Colombia University, Unicef. WASH in Schools Empowers Girls' Education Proceedings of the Menstrual Hygiene Management in Schools Virtual Conference 2012. 2012.

[46] Mason L, Nyothach E, Alexander K, Odhiambo FO, Eleveld A, Vulule J, et al 'We keep it secret so no one should know'—a qualitative study to explore young schoolgirls attitudes and experiences with menstruation in rural western Kenya. PLoS One. 2013;8(11):e79132 10.1371/journal.pone.0079132 ; PubMed Central PMCID: PMCPMC3828248. 24244435 10.1371/journal.pone.0079132 PMC3828248

[47] McMahon SA, Winch PJ, Caruso BA, Obure AF, Ogutu EA, Ochari IA, et al 'The girl with her period is the one to hang her head' Reflections on menstrual management among schoolgirls in rural Kenya. BMC Int Health Hum Rights. 2011;11:7 Epub 2011/06/18. 10.1186/1472-698X-11-7 ; PubMed Central PMCID: PMCPMC3129305. 21679414 10.1186/1472-698X-11-7 PMC3129305

[48] WaterAid. Is menstrual hygiene and management an issue for adolescent school girls? A comparative study of four schools in different settings of Nepal. 2009.

[49] Vallely A, Fitzgerald L, Fiya V, Aeno H, Kelly A, Sauk J, et al Intravaginal practices and microbicide acceptability in Papua New Guinea: implications for HIV prevention in a moderate-prevalence setting. BMC Res Notes. 2012;5:613 10.1186/1756-0500-5-613 ; PubMed Central PMCID: PMCPMC3599571. 23116431 10.1186/1756-0500-5-613 PMC3599571

[50] Mahajan AP, Sayles JN, Patel VA, Remien RH, Sawires SR, Ortiz DJ, et al Stigma in the HIV/AIDS epidemic: a review of the literature and recommendations for the way forward. AIDS. 2008;22 Suppl 2:S67–79. 10.1097/01.aids.0000327438.13291.62 ; PubMed Central PMCID: PMCPMC2835402. 18641472 10.1097/01.aids.0000327438.13291.62 PMC2835402

[51] Alam M, Luby SP, Halder AK, SIslam K, Opel A, Shoab AK, et al Menstrual hygiene management among Bangladeshi adolescent schoolgirls and risk factors affecting school absence: results from a cross-sectional survey. BMJ Open. 2017;7 10.1136/bmjopen-2016-015508 28694347 10.1136/bmjopen-2016-015508 PMC5541609

[52] Oster E, Thornton R. Menstruation, sanitary products, and school attendance: Evidence from a randomized evaluation. American Economic Journal: Applied Economics. 2011:91–100.

[53] Hennegan J, Montgomery P. Do Menstrual Hygiene Management Interventions Improve Education and Psychosocial Outcomes for Women and Girls in Low and Middle Income Countries? A Systematic Review. PLoS One. 2016;11(2):e0146985 Epub 2016/02/11. 10.1371/journal.pone.0146985 ; PubMed Central PMCID: PMCPMC4749306. 26862750 10.1371/journal.pone.0146985 PMC4749306

[54] Ttofi MM, DP F. Bullying: Short-Term and Long-Term Effects, and the Importance of Defiance Theory in Explanation and Prevention. Victims & Offenders. 2008;3(2–3):289–312. 10.1080/15564880802143397

[55] Oster E, Thornton R. Determinants of Technology Adoption: Peer Effects in Menstrual Cup Take-Up. Journal of the European Economic Association. 2012;10(6):1263–93. 10.1111/j.1542-4774.2012.01090.x

